# Automated bone age assessment in a German pediatric cohort: agreement between an artificial intelligence software and the manual Greulich and Pyle method

**DOI:** 10.1007/s00330-023-10543-0

**Published:** 2023-12-28

**Authors:** Daniel Gräfe, Anne Bettina Beeskow, Roland Pfäffle, Maciej Rosolowski, Tek Sin Chung, Matthew David DiFranco

**Affiliations:** 1 Department of Pediatric Radiology, University Hospital, Leipzig, Germany; 2 Department of Pediatrics, University Hospital, Leipzig, Germany; 3 Image Biopsy Lab GmbH, Vienna, Austria

**Keywords:** Bone age measurements, Artificial intelligence, Growth, X-rays, Hand

## Abstract

**Objectives:**

This study aimed to evaluate the performance of artificial intelligence (AI) software in bone age (BA) assessment, according to the Greulich and Pyle (G&P) method in a German pediatric cohort.

**Materials and methods:**

Hand radiographs of 306 pediatric patients aged 1–18 years (153 boys, 153 girls, 18 patients per year of life)—including a subgroup of patients in the age group for which the software is declared (243 patients)—were analyzed retrospectively. Two pediatric radiologists and one endocrinologist made independent blinded BA reads. Subsequently, AI software estimated BA from the same images. Both agreements, accuracy, and interchangeability between AI and expert readers were assessed.

**Results:**

The mean difference between the average of three expert readers and AI software was 0.39 months with a mean absolute difference (MAD) of 6.8 months (1.73 months for the mean difference and 6.0 months for MAD in the intended use subgroup). Performance in boys was slightly worse than in girls (MAD 6.3 months vs. 5.6 months). Regression analyses showed constant bias (slope of 1.01 with a 95% CI 0.99–1.02). The estimated equivalence index for interchangeability was − 14.3 (95% CI −27.6 to − 1.1).

**Conclusion:**

In terms of BA assessment, the new AI software was interchangeable with expert readers using the G&P method.

**Clinical relevance statement:**

The use of AI software enables every physician to provide expert reader quality in bone age assessment.

**Key Points:**

• *A novel artificial intelligence–based software for bone age estimation has not yet been clinically validated.*

• *Artificial intelligence showed a good agreement and high accuracy with expert radiologists performing bone age assessment.*

• *Artificial intelligence showed to be interchangeable with expert readers.*

## Introduction

Bone age (BA) is a widely used index in pediatric endocrinology, orthodontics, and orthopedics for the definition of skeletal maturity. It is defined by the age expressed in years that corresponds to the maturation level of the bones in comparison with the chronological age (CA) of the individual [1]. The images obtained by hand and wrist X-rays reflect the development of the different types of bones in this skeletal group [2, 3], and this information, associated with the characterization of the shape and changes of bone components, represents an important factor in an individual biological development process [4]. BA may be affected by patient sex; nutrition; metabolic, genetic, and social factors; and acute or chronic diseases, including endocrine dysfunction [2, 3, 5, 6].

The most common manual method used for BA assessment is the Greulich and Pyle (G&P) method due to its simplicity and speediness, needing roughly 1.4 min for the evaluation [7, 8]. According to Martin et al [8], it is the preferred procedure by 76% of pediatric endocrinologists and radiologists. Unfortunately, the pitfall of this and other manual bone age estimation methods is the need for more consistency in repeat ratings of the same hand radiograph by one or more readers, as demonstrated by known intra and inter-observer errors [9–11].

With the arrival of digital imaging, numerous attempts have been made to develop image-processing techniques that automatically extract the key morphological features of ossification in the bones to provide a more effective and objective approach to BA assessments [12]. As there is a single image of the left hand and wrist and relatively standardized findings [13, 14], the rapidly evolving deep learning technology world has shown promising results in this field [15].

Recently, a novel, fully automated, Conformité Européenne (CE)–certified radiological image-processing software has been developed to aid medical professionals in the estimation of pediatric BA according to the G&P method using hand radiographs of children aged between 36 and 192 months (females) and 204 months (males). So far, no clinical validation of the software has been published in any literature.

This study aimed to validate the agreement between expert radiologist readers and this new artificial intelligence (AI) software independently from the manufacturer on a cohort of patients from a German population for the age range of intended use of the software as well as for the whole pediatric age range.

## Material and methods

### Patient selection and study design

The local ethics committee granted ethical approval for this retrospective study (EK 46/2020) and the ethics board waived written and informed consent because of the study’s retrospective nature. All methods and procedures were performed following the relevant guidelines and regulations. Furthermore, all retrospectively assessed examinations were performed on an Axiom Aristos FX (Siemens) without a scatter grid and employing a 0.1-mm copper filter. The left hand in posterior-anterior projection was used in all cases for radiography.

A total of 5612 exams of children aged between 1 and 18 (2812 females) were available on the radiological database system (dated 2011–2020). Stratified random sampling was performed to select nine patients for each year of life and sex, resulting in a study sample of 306 patients (153 females and 153 males). The exclusion criterion was poor image quality or positioning, hindering manual estimation of G&P BA.

Based on the clinical relevance and the intended use of the AI software IB Lab PANDA, we assessed the sex-specific performance of a subpopulation of patients with CAs from 36 to 192 months (females) and 36 to 204 months (males).

### Artificial intelligence model for automated bone age assessment

The AI model for estimating BA from hand radiographs was implemented into the CE-marked, commercially available software IB Lab PANDA (version 1.99), which automates hand radiograph assessment according to the G&P method. Since no scientific literature on the development of this AI software has been published so far, a short outline of this software is provided below: Training of the AI was performed on a dataset consisting of over 12,000 anonymized hand radiographs drawn from a population of US patients from the clinical workflow (54% male, 46% female, mean patient age of 127 months). These images were interpreted by pediatric radiologists, who documented skeletal age in the radiology report based on a visual comparison to the G&P atlas [3].

**Fig. 1:**
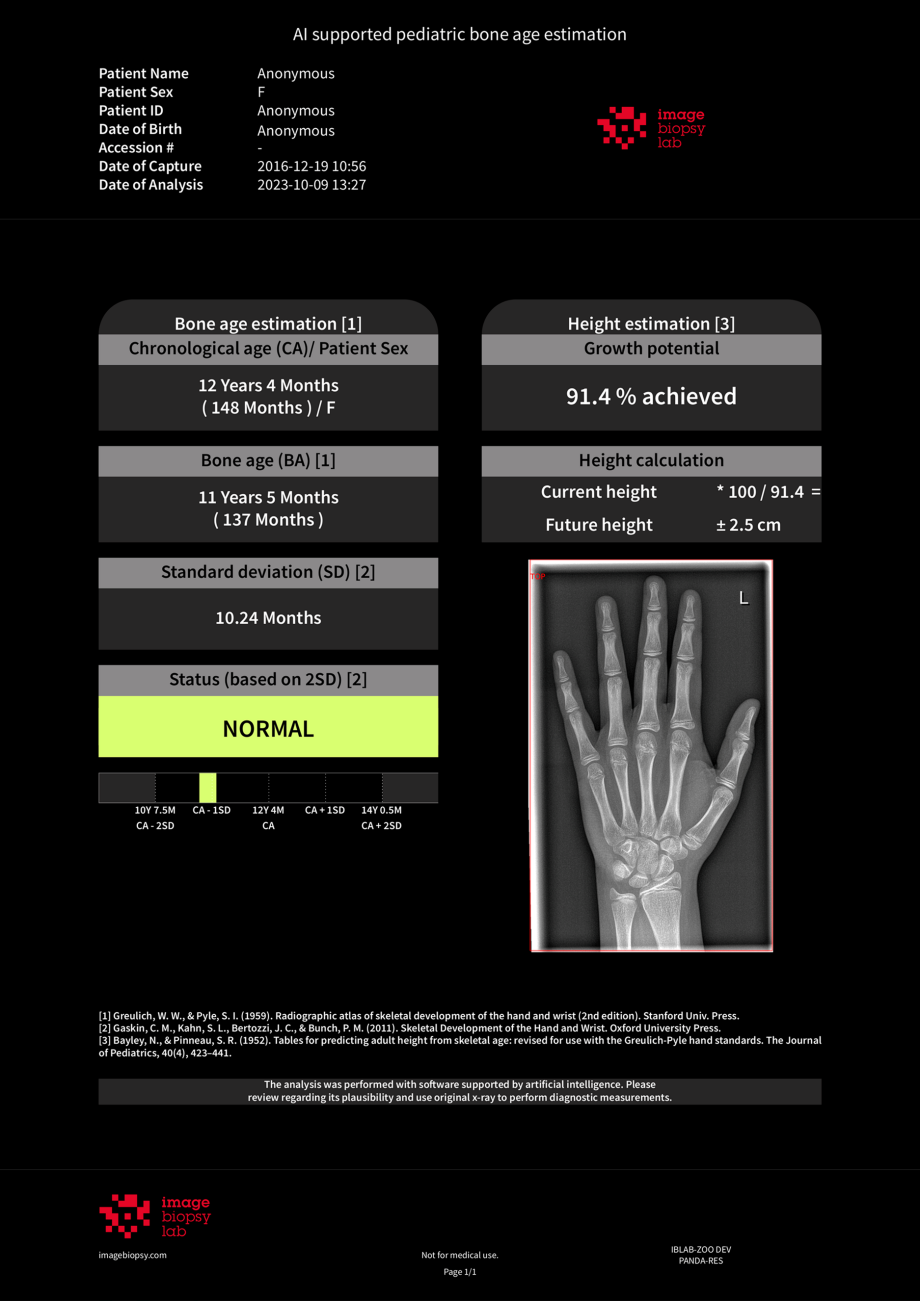
PANDA report file of a girl with a chronological age of 12.4 years. The bone age predicted by the software was 11.4 years, matching the manual Greulich and Pyle method applied by expert readers

IB Lab PANDA v1.13.21 has an intended use population based on CA of girls aged 36 to 192 months and boys aged 36 to 204 months, with CA based on the date of radiograph acquisition. IB Lab PANDA generates a graphical report with BA to the nearest month and a secondary capture of the input radiograph for visual inspection that designates the region of the image, which was used for analysis by the software (Fig. 1).

Automated AI analysis of the radiographic images was carried out via an internal clinical pipeline by installation on a dedicated standalone PC within a Docker container, configured as a Picture Archive and Communication System sending and receiving node.

### Expert reader bone age assessment

All radiographs were analyzed independently in separate sessions by three physicians with different levels of expertise (D.G. pediatric radiologist with 7 years of experience; A.B. pediatric radiologist with 4 years of experience; and R.P. pediatric endocrinologist with 30 years of experience in pediatric BA determination) using the G&P atlas [3]. In cases of discrepancy between hand and wrist BA, the maturity of the forehand was preferred [16]. The ground truth was established using the average of the three experts.

### Statistical analysis

Statistical analysis was performed with RStudio (Version 2023.06.2, PBC).

The agreement between the automated AI assessment and ground truth was quantified by mean absolute difference (MAD) and root mean square error (RMSE). Bland-Altman analysis was employed for the mean difference and limits of agreement. Pearson’s product-moment correlation was evaluated between AI and ground truth. The intraclass correlation coefficient (ICC) assessed the agreement between the three readers and between AI and ground truth. Pearson’s correlation coefficient was employed to correlate AI bone age and expert readers.

Furthermore, interchangeability between BA assessments by AI and expert readers was evaluated by determining the equivalence index *γ* [17, 18]. For use with multiple readers in this study, the calculation was modified such that all measurements came from one reader that scored each patient’s images three times using the reference method (G&P atlas) and once using a novel method (AI software). The confidence interval (CI) was estimated by drawing 10,000 random samples. An equivalence index *γ* > 0 would mean that the deviation between AI and the assessment from the readers is larger than the deviation among the assessments from the readers. *γ* ≤ 0 indicates that AI is interchangeable with expert readers.

## Results

After stratified sampling of 5612 exams, none of the resulting 306 studies met the exclusion criteria. Thus, a total of 306 radiograph images of German children performed between 2011 and 2020 were included in the final study cohort and analyzed. The demographics of the study population are shown in Table 1.

**Table 1:** Demographic data of the study population

	Study population
Patients (no.)	306
Sex	153 female
Age (y)	9.5 (± 4.9)
Height (cm)	131.1 (± 30.0)
Weight (kg)	35.3 (± 23.2)
BMI (kg/m^2^)	18.3 (± 6.3)

*BMI* body mass index

The underlying diseases and indications for this specific pediatric cohort were presented as follows:

- 84.3% (*n* = 258) with endocrine, nutritional, and metabolic diseases, such as small stature, high stature, or hypopituitarism.
- 5.2% (*n* = 16) have chromosomal anomalies such as Turner syndrome.
- 4.9% (*n* = 15) have mental and neurologic disorders, such as developmental delay.
- 3.6% (*n* = 11) had growth disturbances due to medication side effects.
- 2.0% (*n* = 6) with perinatal conditions, such as intrauterine growth retardation.

The ethnic background was Caucasian in 294 (96%) children and not available retrospectively in 12 (4%) children.

**Fig. 2:**
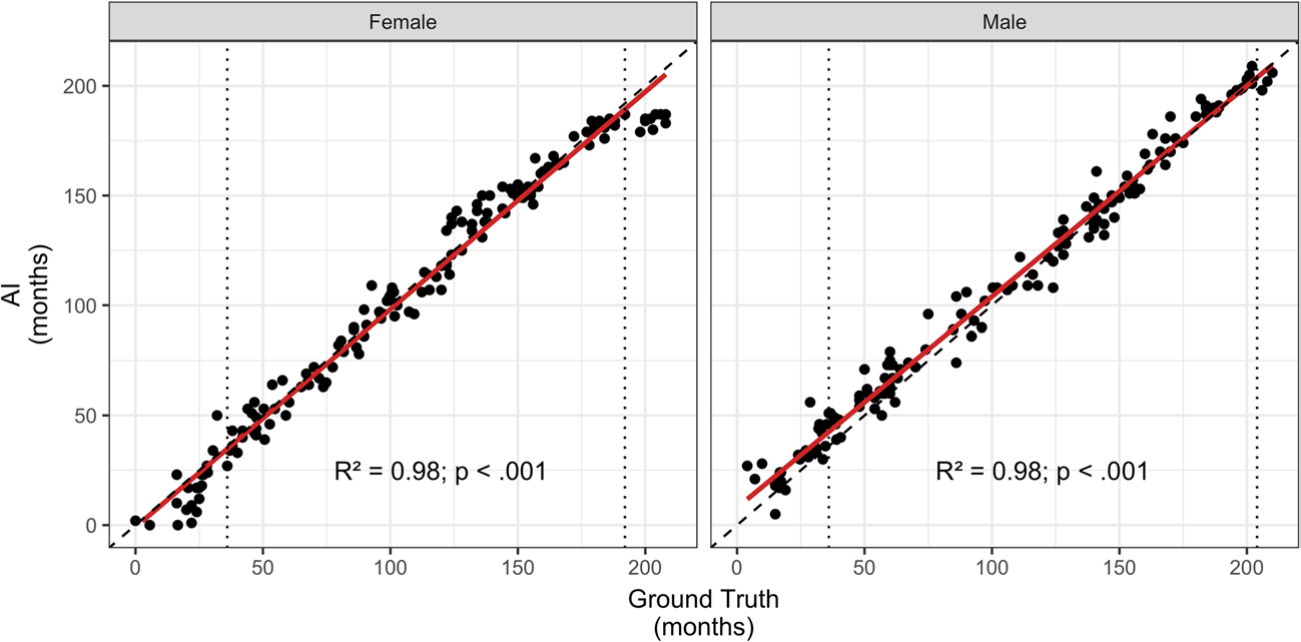
Regression analysis between the ground truth (mean assessment of readers) and artificial intelligence (AI) of the entire cohort. The 95% confidence interval for the slope (0.99 for females and 1.03 for males) of the regression (red line) includes the identity function with slope = 1.00 (dashed line). Dotted lines: age limits for intended use of the AI software

The automated AI software analyzed all radiograph images in the cohort without rejecting any. Correlation analysis showed a strong correlation between BA as determined by AI and ground truth (*r* (304) = .98, *p* < .001). Linear regression revealed constant bias (slope 1.01 with a 95% CI 0.99–1.02) (Fig. 2). The mean difference between the AI and ground truth in the cohort was 0.45 months, the MAD 6.8 months, and the RMSE 9.0 months.

**Fig. 3:**
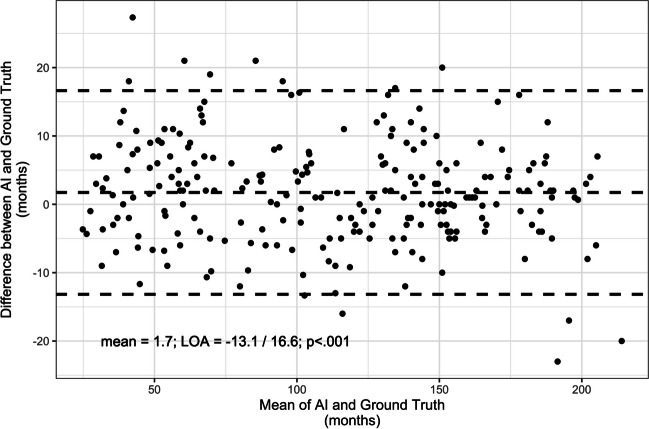
The Bland-Altman plot demonstrates excellent agreement between artificial intelligence (AI) and the mean assessment of observers on the cohort restricted to the intended use of the AI software. Dashed lines: mean difference, upper limits of agreement and lower limits of agreement

Subsequently, according to the age range of intended use of the AI software, further cohort analysis was restricted to girls aged 36 to 192 months and boys aged 36 to 204 months, resulting in 243 patients (125 boys and 118 girls). Bland-Altman analysis showed consistent, small bias with a mean difference of 1.73 months (95% CI 0.8–2.7,* p* < 0.001) between the mean differences of both methods for BA determination (Fig. 3), and the residuals between AI BA and ground truth exhibited a normal distribution (*p* = 0.34 for Shapiro-Wilk). For the cohort within the age range of intended use of the AI software, differences between AI and ground truth were 1.7 months for mean error, 6.0 months for MAD, and 7.8 months for RSME (Table 2).

**Table 2:** AI model versus the mean of the three expert readers in the cohort restricted to the intended use of the AI software. The negative equivalence index indicates that AI and expert readers are interchangeable

	Sample	Mean difference	MAD	RSME	Lower/upper LOA	Equivalence Index
Total	*n* = 243	1.7 [0.8–2.7]	6.0	7.8	− 13.1 / 16.6	− 14.3 [− 27.6, − 1.1]
Boys (3 to 17 years)	*n* = 125	3.4 [2.0–4.7]	6.3	8.3	− 11.5 / 18.3	− 6.9 [− 26.0, 12.7]
Girls (3 to 16 years)	*n* = 118	− 0.1 [− 1.4 to 1.3]	5.6	7.1	− 14.2 / 14.1	− 22.2 [− 40.4, − 4.0]

*AI* artificial intelligence, *LOA* Bland-Altman limits of agreement, *MAD* mean absolute difference, *RSME* root squared mean error

[-]: 95% confidence interval

Inter-rater agreement, as measured by ICC, was excellent between the three readers (ICC 0.98 [CI 0.97–0.98]) and between AI software and ground truth (ICC 0.99 [0.98–0.99]). The equivalence index *γ* was < 0, indicating interchangeability between human readers and AI for both boys and girls.

## Discussion

This retrospective study evaluated the agreement between a novel AI software developed for automated BA assessment in a German pediatric cohort and the manual G&P method used by independent expert readers. This validation study is the first to be published for this specific AI software.

The results demonstrated that the novel AI software allowed for a highly accurate BA assessment by automatically analyzing radiographs of the left hand compared to the G&P method. Results showed excellent agreement between the AI-based and experienced expert reader assessments, which served as the reference standard in this study. Importantly, this agreement was achieved regardless of the patient’s sex.

The deviation between PANDA BA and ground truth, a MAD of 6.0 months and RSME of 7.8 months, is within the recently reported range of human readers [19]. This is supported by our finding that PANDA BA is interchangeable with human readers, employing the individual equivalence index by Obuchowski [17].

Besides IB Lab PANDA, the authors are aware of three other commercially available, CE-certified AI-based solutions for bone age determination: Visiana BoneXpert (Visiana), Vuno Med-BoneAge by VUNO (VUNO), and Gleamer BoneView Bone age (Gleamer). Although benchmarking the performance of these programs was not the aim of this study, the results of PANDA can be compared for the first time with the results of other previously established AI programs as reported in the literature:

Recently, Booz et al reported a MAD of 4.1 months and a RSME of 4.6 months for BoneXpert, the longest-standing bone age software [20]. Kim et al reported a RSME between VUNO and expert readers of 7.2 months. For Gleamer, the newest AI software, Nguyen et al calculated a MAD of 5.9 months [21].

Of interest, the software was able to analyze all radiographs without any rejections automatically, which implies a high level of efficiency similar to that obtained by other studies investigating BA automated assessments in similar patient populations, reinforcing the reliability and efficiency of this novel software in clinical routine [20].

The G&P method is a well-established, widely available, and cost-effective approach for BA assessment. However, it has the inherent disadvantage of a high dependence on the radiologist’s or physician’s expertise, which leads to inter-rater and intra-rater variability [10, 11, 19]. In 2019, Dallora et al [12] conducted a systematic literature review and meta-analysis. They provided evidence that suggested a trend towards automating BA assessment aimed at reducing the dependability upon human input and reducing the subjectivity of the traditional BA assessment methods [22].

There are several limitations to this study. Due to the retrospective design of the trial, only radiographs from one institution were evaluated, with only 306 patients analyzed. As such, to re-evaluate the results and conclusions of our single-center study, a multicenter study with a larger patient cohort would be necessary. Based on the results in the regression plot (Fig. 2), we can see a loss of accuracy for patients under 3 years and older than 16 years. However, the G&P method is known to be the most appropriate for children older than 3 years up until the end of puberty [1]. Inaccuracies in the older age group can be explained by similarities in the bone structure of late adolescents resulting in discrepancies in BA assessment. At this stage, most of the hand bone structures are fully developed, and skeletal maturity is assessed mainly based on the extent of epiphyseal fusion of the ulna and radius [2].

Furthermore, the AI software functions as a “black box” [23], indicating that the elements of the X-ray image that influenced the estimation of the bone age are not explicitly explained. In practical terms, this is not a problem with a properly performing algorithm. However, the quality of the input X-ray image must be verified properly by a human reader.

Finally, the value of a validation study of another AI-assisted bone age software could be questioned as there are already proper applications commercially available. Each program, however, has its advantages and disadvantages. For example, a more attractive pricing model might be more critical for some institutions than an increased accuracy of bone age determination of 1 month. However, a vendor-independent peer-reviewed validation, such as in the present study, is indispensable for an informed decision in favor of one or the other software.

In conclusion, this study demonstrated that a novel AI software is interchangeable with human expert readers and enables a highly accurate BA assessment, regardless of patient sex, in a German patient cohort compared to the manual G&P method.

## Abbreviations

AI: Artificial intelligence
BA: Bone age
CA: Chronological age
CE: Conformité Européenne
CI: Confidence interval
G&P: Greulich and Pyle
ICC: Intraclass correlation coefficient
LOA: Limits of agreement
MAD: Mean absolute difference
RSME: Root mean square error
SD: Standard deviation

## Declarations

### Guarantor

The scientific guarantor of this publication is Daniel Gräfe.

### Statistics and biometry

One of the authors (Maciej Rosolowski) has significant statistical expertise.

### Study subjects or cohorts overlap

No study subjects or cohorts have been previously reported.

### Methodology

• retrospective

• cross-sectional study

• performed at one institution
